# Comparison of Culture and Rapid Enzyme Immunoassay for the Detection of Group B Streptococcus in High-Risk Pregnancies

**DOI:** 10.1155/S1064744994000499

**Published:** 1994

**Authors:** Mara J. Dinsmoor, Harry P. Dalton, Thomas C. C. Peng, James T. Christmas, Sousan Sayahtaheri-Altaie, Kevin Harvey, J. Peter VanDorsten

**Affiliations:** Departments of Obstetrics and Gynecology and Pathology Medical College of Virginia/Virginia Commonwealth University Box 980034 MCV Station Richmond VA 23298 USA

## Abstract

*Objective:* The purpose of this study was to evaluate the Equate Strep B® test for clinical use in patients at high risk for complications from group B streptococcus (GBS) disease.

*Methods:* Vaginoperineal swabs were obtained from patients with preterm premature rupture of the membranes and/or preterm labor and semiquantitative GBS cultures and Equate® assay were performed.

*Results:* From May 14, 1990, to April 30, 1992, 650 patients were enrolled; 626 had both culture and Equate® results available, of whom 24% were colonized with GBS. The sensitivity, specificity, positive predictive value, and negative predictive value of the rapid assay were 28%, 84%, 35%, and 79%, respectively. Although the prevalence of GBS was higher in patients with ruptured membranes compared with those with intact membranes, rupture of membranes did not affect test sensitivity or specificity.

*Conclusions:* We conclude that the Equate® rapid assay is not a sensitive method of GBS detection in high-risk patients.


Infectious Diseases in Obstetrics and Gynecology 2:115–119 (1994)
© 1994 Wiley-Liss, Inc.

### Comparison of Culture and Rapid Enzyme Immunoassay for the Detection of Group B Streptococcus in High-Risk Pregnancies

**Mara J. Dinsmoor, Harry P. Dalton, Thomas C.C. Peng, James T. Christmas, Sousan Sayahtaheri-Altaie, Kevin Harvey, and J. Peter VanDorsten**

*Departments of Obstetrics and Gynecology and Pathology, Medical College of Virginia/Virginia Commonwealth University, Richmond, VA*

### Abstract

***Objective:*** **The purpose of this study was to evaluate the Equate Strep B® test for clinical use in patients at high risk for complications from group B streptococcus (GBS) disease.**

***Methods:*** **Vaginoperineal swabs were obtained from patients with preterm premature rupture of the membranes and/or preterm labor and semiquantitative GBS cultures and Equate® assay were performed.**

***Results:*** **From May 14, 1990, to April 30, 1992, 650 patients were enrolled; 626 had both culture and Equate® results available, of whom 24% were colonized with GBS. The sensitivity, specificity, positive predictive value, and negative predictive value of the rapid assay were 28%, 84%, 35%, and 79%, respectively. Although the prevalence of GBS was higher in patients with ruptured membranes compared with those with intact membranes, rupture of membranes did not affect test sensitivity or specificity.**

***Conclusions:*** **We conclude that the Equate® rapid assay is not a sensitive method of GBS detection in high-risk patients.** © 1994 Wiley-Liss, Inc.

Key words

Rapid diagnostic tests, maternal colonization, neonatal colonization

Vaginal colonization with group B streptococcus (GBS) occurs in 10–30% of normal pregnancies. Although maternal disease due to GBS colonization is relatively rare, neonatal colonization occurs in 40–70% of infants born to colonized women. Approximately 1–2% of colonized infants ultimately develop early onset neonatal GBS sepsis, a disease with a high mortality rate and much morbidity. Factors that increase the risk for development of GBS sepsis include low birth weight and prematurity, prolonged membrane rupture, and maternal factors such as fever in labor and heavy vaginal colonization.

It has been shown that intrapartum administration of ampicillin to mothers colonized with GBS in the 3rd trimester results in a decrease in neonatal colonization and neonatal GBS sepsis.[1,2] The problem remains, however, in identifying those patients who would most benefit from ampicillin prophylaxis. Antepartum cultures are not predictive of intrapartum colonization status, as only 55–67% of patients with positive cultures in the 3rd trimester will have positive cultures at the time of labor, even in the absence of antibiotic treatment.[3,4] Moreover, approximately 5–10% of patients with negative antepartum cultures will be colonized when

Address correspondence/reprint requests to Dr. Mara J. Dinsmoor, Box 980034 MCV Station, Richmond, VA 23298.

Received March 8, 1994

Accepted May 24, 1994

Clinical Study

**TABLE 1. Sensitivity, specificity, and predictive value of Equate® rapid GBS detection kit in comparison with selective GBS culture**

| | All patients (N = 626) | Intact membranes (N = 394) | Ruptured membranes (N = 232) | Heavily colonized (N = 626) |
|---|---|---|---|---|
| Sensitivity | 28% | 28% | 27% | 33% |
| Specificity | 84% | 82% | 88% | 83% |
| PPV | 35% | 27% | 51% | 21% |
| NPV | 79% | 83% | 72% | 91% |
| Prevalence | 24% | 19% | 32% | 12% |

evaluated intrapartum.[3,4] Clearly, there is a need for the ability to rapidly and accurately detect the presence of GBS, especially in those patients at high risk for neonatal GBS sepsis.

Our purpose was to evaluate one of the currently available rapid test kits for clinical use in a population at high risk for maternal GBS colonization and the subsequent development of neonatal GBS colonization and sepsis.

### SUBJECTS AND METHODS

As part of an ongoing study of the use of a rapid GBS test in the management of high-risk pregnancies, all women admitted to the Medical College of Virginia Hospital with the diagnosis of preterm (<37 complete weeks gestation) labor and/or preterm premature rupture of the membranes (PTROM) were offered enrollment in the study. In addition, those patients who were undergoing a preterm induction of labor for maternal indications (usually severe preeclampsia) were eligible. Exclusion criteria included age <18 years, antibiotic use in the previous 2 weeks, or a previous infant with GBS sepsis. The study was approved by the Committee on the Conduct of Human Research at Virginia Commonwealth University, and an informed consent was obtained from all patients.

Three vaginoperineal swabs were obtained from all study patients at the time of admission to labor and delivery. Two swabs were transported in culture media to the clinical microbiology laboratory, where 1 was streaked onto non-selective medium (5% sheep blood agar) and the other was placed in selective enrichment broth (Todd-Hewitt broth supplemented with 5% sheep blood, 0.01 mg/ml of colistin, and 0.005 mg/ml of oxolinic acid). Following 24 h of incubation, the broth was plated, again on non-selective medium. Colonies of GBS were confirmed using Phadebact® Strep B latex agglutination test (Remel, Lenexa, KS), in accordance with the manufacturer's recommended procedures.

The 3rd swab, provided in the Equate® kit, was used to perform the Equate® assay, as per the manufacturer's directions. Following an initial 11-month trial period during which 37 assays were performed by 3rd-year house officers, all Equate® assays were performed by 1 of 4 designated laboratory technicians.

Results of the culture for GBS were reported semiquantitatively as containing many, moderate, or rare colonies or as negative based on the number of quadrants on the plate that contained growth. Those samples that resulted in growth only following broth incubation were classified as containing only rare colonies. Equate® assays were reported as positive or negative.

Chi-square analysis was used to compare discrete variables. $P < 0.05$ was considered significant.

### RESULTS

Between May 14, 1990, and April 30, 1992, 650 patients were enrolled in the study. Of these, 626 had both Equate® and vaginal culture results available. The mean age of the study participants was 24.6 ± 5.7 years and the mean gestational age at enrollment was 32.2 ± 3.3 weeks. Thirty-five percent of the patients were nulliparous, while 21% had a history of preterm birth. Three hundred twenty-eight (52%) of the patients were admitted with preterm labor, 231 (37%) with PTROM, and 67 (11%) were enrolled at the time of preterm induction. All patients in the latter group had intact membranes at enrollment.

One hundred forty-nine (24%) of the patients were culture positive for GBS. Of these, 77 (52%) had rare, 54 (36%) had moderate, and 18 (12%) had many colonies. Table 1 illustrates the results of the Equate® assay compared with culture.

**TABLE 2. Summary of performance in clinical settings of different antigen detection tests for GBS in comparison with culture**

| Reference | Assay type | Patient population | GBS Prevalence | Sensitivity | Specificity | PPV | NPV |
|---|---|---|---|---|---|---|---|
| Towers et al.[15] | EIA[a] (Equate®) | Antepartum N = 131 | 12% | 60% | 92% | 57% | 93% |
| Skoll et al.[b] | EIA (Equate®) | Laboring N = 1,052 | 9% | 15% | 99% | 67% | 92% |
| Greenspoon et al.[5] | EIA (Equate®) | Laboring N = 250 | 2% | 33% | 99% | 40% | 98% |
| Gentry et al.[7] | EIA (ICON®) | Laboring N = 300 | 10% | 33% | 95% | 24% | 93% |
| Armer et al.[8] | EIA (ICON®) | Laboring N = 182 | 23% | 11%[c] | 100% | 100% | 78% |
| Isada and Grossman[12] | LA[b] (Bactigen®) | Laboring N = 431 | 4% | 58% | 93% | 28% | 98% |
| Brady et al.[14] | LA (Directigen®) | Laboring N = 500 | 5% | 88% | 100% | 92% | 99% |
| Clark et al.[10] | LA (Directigen®) | Laboring N = 314 | 29% | 30%[c] | 93% | 63% | 76% |
| Green et al.[13] | LA | Laboring N = 4,251 | 5% | | | | |
| | (Directigen®) | | | 24% | 99% | 81% | 96% |
| | (Bactigen®) | | | 20% | 99% | 79% | 96% |
| Greenspoon et al.[5] | LA (Bactigen®) | Laboring N = 250 | 2% | 33% | 95% | 15% | 98% |
| Skoll et al.[6] | LA (Streptex®) | Laboring N = 1,060 | 9% | 15% | 99% | 67% | 92% |
| Kontnick and Edberg[11] | LA (Streptex®) | Antepartum N = 434 | 15% | 19%[c] | 100% | 92% | 88% |

[a]EIA = enzyme immunoassay.
[b]LA = latex agglutination.
[c]Compared with GBS culture on selective medium.

When patients with intact membranes were analyzed separately from those with PTROM, the results were similar. Although the sensitivity and specificity were essentially the same, the positive predictive value (PPV) was higher and the negative predictive value (NPV) was lower in those patients with PTROM. This finding is not surprising, given the significantly higher prevalence of GBS colonization in the patients with PTROM (32% vs. 19%, $P = 0.004$).

Because prior studies of rapid GBS detection systems have shown improved sensitivity when applied only to those patients with heavy vaginal colonization,[5–13] we then analyzed our results considering only those patients with heavy colonization, defining moderate or many colonies (≥2 quadrants on the initial plate or >100,000 cfu/ml) as positive (N = 72). As shown in Table 1, the sensitivity and NPV of the rapid test also improved in our study, although marginally, when only heavy maternal colonization was considered. The false negative rate, or the percentage of carriers not identified, increases with decreasing colony counts. Thirteen percent of those with rare colonies, 8% of those with moderate colonies, and 3% of women with many colonies were not detected by the rapid test.

### DISCUSSION

Although the first studies to utilize rapid detection tests reported high sensitivities for the detection of GBS (58% by Isada and Grossman[12] and 88% by Brady et al.[14]), these results have been criticized for comparing the rapid tests with culture results obtained with non-selective medium. Subsequent studies of these particular rapid tests as well as others have reported much lower sensitivities (Table 2).

Our finding that the sensitivity of the Equate® rapid enzyme immunoassay is low is in agreement with other investigators, including those who have used non-selective culture medium to detect GBS colonization. Although using selective medium for comparison could result in lower sensitivities, we optimized sensitivity by limiting the number of personnel who performed the test, using the swab supplied with the Equate® kit, using reagents at room temperature, and performing the test immediately, following the manufacturer's directions exactly. Although one group[15] reported a sensitivity of 60% and specificity of 92% for the Equate® assay in antepartum patients, other studies in laboring patients have reported much lower sensitivities of 22% and 33%.[5,6] It is possible that there are substances present in the vagina of laboring women that might interfere with the rapid test and partially explain the lower sensitivities seen in these patients.

Results with other rapid test methods have proven equally disappointing. Using a different immunoenzyme assay (ICON Strep B®), Gentry et al.[7] reported a sensitivity of 33%, and Armer et al.[8] reported a sensitivity of 11%. Both authors conclude that the assay is not sufficiently sensitive in detecting light colonization, precluding routine use in the clinical setting. Additional studies using latex agglutination tests have resulted in similar findings, with sensitivities ranging from 15 to 30% and specificities from 93 to 100%, with NPVs of 76–88% in those populations with significant colonization rates.[5,6,10–14]

Using yet another enzyme immunoassay (Quidel®), Granato and Petosa[9] reported a sensitivity of 89%, a specificity of 99%, and an NPV of 99% in comparison with culture on non-selective medium. However, when lightly colonized women are included, the sensitivity decreases to 74%. It remains to be seen whether this rapid test will be adequately sensitive when compared with culture using selective medium.

Although many investigators have found increased sensitivity when the rapid tests were applied only to heavily colonized women, whether detection of only heavily colonized mothers is adequate is a matter of some discussion.[8,10,16] Isada and Grossman[12] found that, in a population with a GBS prevalence of 4.4%, all 9 cases of "clinically significant" perinatal GBS morbidity were identified using a rapid latex agglutination test, including 5 cases of neonatal GBS sepsis. Although heavy maternal colonization increases the risk for neonatal GBS sepsis, many cases of neonatal GBS sepsis have been reported following delivery of women who were only lightly colonized.[2,8,15–17]

A prospective study by Tuppurainen and Hallman[18] found that using a latex agglutination test (Streptolatex, Orion Diagnostica, Finland) for selecting those intrapartum patients to be randomized to receive prophylactic penicillin or placebo resulted in neonatal GBS disease in 0.07% of the rapid-test negative (untreated) group. Nine percent of the neonates born to rapid-test positive patients who received placebo developed GBS disease, while 1.1% of the neonates born to treated women had GBS disease. In a similar study, Morales and Lim[17] performed rapid tests on 260 patients with PTROM. Eighty-four patients had positive rapid tests, 36 of whom were given antibiotic prophylaxis prior to delivery. Although patients with positive rapid tests who were untreated were more likely to develop chorioamnionitis (13% vs. 0) and to deliver neonates who developed neonatal sepsis (15% vs. 0), no results are reported for those women who were rapid-test negative. As a result, it is impossible to evaluate the effectiveness of this treatment protocol.

Unless chemoprophylaxis protocols based on the use of rapid assays for the intrapartum detection of maternal GBS colonization can be shown to result in similar or lower neonatal GBS colonization and sepsis rates compared with current perhaps less selective management protocols,[19] the use of the Equate® assay in the intrapartum management of high-risk pregnancies is to be discouraged.

### ACKNOWLEDGMENTS

This work was funded in part by an A.D. Williams Grant-in-Aid from the Medical College of Virginia/Virginia Commonwealth University and the Stuart Pharmaceutical Fellowship of the Infectious Disease Society for Obstetrics-Gynecology.

### REFERENCES

1. Boyer KM, Gotoff SP: Prevention of early-onset neonatal group B streptococcal disease with selective intrapartum chemoprophylaxis. N Engl J Med 314:1665–1669, 1986.
2. Morales WJ, Lim DV, Walsh AF: Prevention of neonatal group B streptococcal sepsis by use of a rapid screening test and selective intrapartum prophylaxis. Am J Obstet Gynecol 155:979–983, 1986.
3. Boyer KM, Gadzala CA, Kelly PD, Burd LI, Gotoff SP:

Selective intrapartum chemoprophylaxis of neonatal group B streptococcal early-onset disease. II. Predictive value of prenatal cultures. J Infect Dis 148:802–809, 1983.
4. Allardice JG, Baskett TF, Seshia MMK, et al.: Perinatal group B streptococcal colonization and infection. Am J Obstet Gynecol 142:617–620, 1982.
5. Greenspoon JS, Fishman A, Wilcox JG, Greenspoon RL, Lewis W: Comparison of culture for group B streptococcus versus enzyme immunoassay and latex agglutination rapid tests: Results in 250 patients during labor. Obstet Gynecol 70:97–100, 1991.
6. Skoll MA, Mercer BM, Baselski V, Gray JP, Ryan G, Sibai BM: Evaluation of two rapid group B streptococcal antigen tests in labor and delivery patients. Obstet Gynecol 77:322–326, 1991.
7. Gentry YM, Hillier SL, Eschenbach DA: Evaluation of a rapid enzyme immunoassay test for detection of group B streptococcus. Obstet Gynecol 78:387–401, 1991.
8. Armer T, Clark P, Duff P, Saravanos K: Rapid intrapartum detection of group B streptococcal colonization with an enzyme immunoassay. Am J Obstet Gynecol 168:39–43, 1993.
9. Granato PA, Petosa MT: Evaluation of a rapid screening test for detecting group B streptococci in pregnant women. J Clin Microbiol 29:1536–1538, 1991.
10. Clark P, Armer T, Duff P, Davidson K: Assessment of a rapid latex agglutination test for group B streptococcal colonization of the genital tract. Obstet Gynecol 79:358–363, 1992.
11. Kontnick CM, Edberg SC: Direct detection of group B streptococci from vaginal specimens compared with quantitative culture. J Clin Microbiol 28:336–339, 1990.
12. Isada NB, Grossman JH: A rapid screening test for the diagnosis of endocervical group B streptococci in pregnancy: Microbiologic results and clinical outcome. Obstet Gynecol 70:139–141, 1987.
13. Green M, Dashefsky B, Wald ER, Laifer S, Harger J, Guthrie R: Comparison of two antigen assays for rapid intrapartum detection of vaginal group B streptococcal colonization. J Clin Microbiol 31:78–82, 1993.
14. Brady K, Duff P, Schilhag JC, Herd M: Reliability of a rapid latex fixation test for detecting group B streptococci in the genital tract of parturients at term. Obstet Gynecol 73:678–681, 1989.
15. Towers CV, Garite TJ, Friedman WW, Pircon RA, Nageotte MP: Comparison of a rapid enzyme-linked immunosorbent assay test and the gram stain for detection of group B streptococcus in high-risk antepartum patients. Am J Obstet Gynecol 163:965–967, 1990.
16. Yancey MK, Armer T, Clark P, Duff P: Assessment of rapid identification tests for genital carriage of group B streptococci. Obstet Gynecol 80:1038–1047, 1992.
17. Morales WJ, Lim D: Reduction of group B streptococcal maternal and neonatal infections in preterm pregnancies with premature rupture of membranes through a rapid identification test. Am J Obstet Gynecol 157:13–16, 1987.
18. Tuppurainen N, Hallman M: Prevention of neonatal group B streptococcal disease: Intrapartum detection and chemoprophylaxis of heavily colonized parturients. Obstet Gynecol 73:583–587, 1989.
19. Minkoff H, Mead P: An obstetric approach to the prevention of early-onset group B beta-hemolytic streptococcal sepsis. Am J Obstet Gynecol 154:973–977, 1986.
